# Integrating Fiber Optic Data in Numerical Reservoir Simulation Using Intelligent Optimization Workflow

**DOI:** 10.3390/s20113075

**Published:** 2020-05-29

**Authors:** Giuseppe Feo, Jyotsna Sharma, Stephen Cunningham

**Affiliations:** 1 Department of Petroleum Engineering Louisiana State University, Baton Rouge, LA 70803, USA; gfeo1@lsu.edu; 2 Vaquero Energy, Santa Maria, CA 93454, USA; scunningham@vaqueroenergy.com

**Keywords:** distributed fiber optic sensing, intelligent optimization algorithm, numerical reservoir simulation, enhanced oil recovery, cyclic steam stimulation, automated history match

## Abstract

A novel workflow is presented for integrating fiber optic Distributed Temperature Sensor (DTS) data in numerical simulation model for the Cyclic Steam Stimulation (CSS) process, using an intelligent optimization routine that automatically learns and improves from experience. As the steam–oil relationship is the main driver for forecasting and decision-making in thermal recovery operations, knowledge of downhole steam distribution across the well over time can optimize injection and production. This study uses actual field data from a CSS operation in a heavy oil field in California, and the value of integrating DTS in the history matching process is illustrated as it allows the steam distribution to be accurately estimated along the entire length of the well. The workflow enables the simultaneous history match of water, oil, and temperature profiles, while capturing the reservoir heterogeneity and the actual physics of the injection process, and ultimately reducing the uncertainty in the predictive models. A novel stepwise grid-refinement approach coupled with an evolutionary optimization algorithm was implemented to improve computational efficiency and predictive accuracy. DTS surveillance also made it possible to detect a thermal communication event due to steam channeling in real-time, and even assess the effectiveness of the remedial workover to resolve it, demonstrating the value of continuous fiber optic monitoring.

## 1. Introduction

All industry research indicates that conformance control of steam is important for an efficient thermal enhanced oil recovery process. Ensuring uniform steam chest development optimizes steam oil ratios (SOR), by minimizing steam breakthrough and maximizing reservoir heating and oil production [1]. Once a sufficient understanding of the downhole steam distribution is achieved, flow control devices (FCDs) and isolation devices (such as packers) can be installed in the wellbore to engineer the improvement of steam conformance. However, they do not ensure an acceptable steam conformance since steam conformance can be affected by a number of factors such as reservoir rock and fluid properties, well geometry, completion architecture, heat transfer mechanisms, flow regimes, completion damages, and even the production and injection in surrounding wells. Monitoring the downhole steam distribution profile using fiber optic DTS provides high-resolution datasets through the life of the project, which enables a more comprehensive approach to optimizing steam conformance and SOR. Insufficient injection results in lost production; conversely, excessive injection results in steep fuel costs, wasted heat in the casing, sanding problems in producers, premature equipment failures, decreased reliability of well and surface systems, early steam breakthrough in the formations, and even surface eruptions. Thus, steam conformance monitoring and control are critical for the success of a thermal recovery project.

Monitoring the continuous systematic temperature changes would be the ultimate method to optimize injection and production; however, achieving this through conventional surveillance is challenging. Permanent gauges are unreliable at elevated field temperatures and often require frequent calibrations. Gauges are inherently unable to provide a full steam profile as they can only provide measurement at limited number of locations. Wireline logging has been the proven cost-efficient solution, but in many cases, running a log in a producer or injector would require pulling the tubing pump, which may not always be feasible. Furthermore, the periodicity of logging data is limited to the number of wells to be logged and the number of logging units available. Fiber optic surveillance has proven to be the technology to overcome these challenges as it can be installed on the tubing or casing on either a permanent or semipermanent basis and provide distributed measurements over the entire length of the well continuously in real-time. However, a key challenge in using this emerging technology is the vast amount of data it generates [2]. The need to process exceedingly large data records, often in real-time, makes the usage of automated workflows very appealing for this application [3,4]. In this paper, field data from a heavy oil field in California is analyzed to demonstrate the application of DTS for estimating the downhole steam injection profile, through an automated history matching workflow, leveraging an evolutionary optimization routine.

### 1.1. Distributed Fiber Optic Sensing Technology

Because of the demands for chemical and electrical passivity, as well as operational robustness in the high pressure and high temperature, hostile environments typical of steam operations, distributed fiber optic sensing methods are an excellent way of downhole monitoring along the entire length of the well [5]. The methods of fiber optic sensing are relatively easy to adapt for use in new or existing wells either permanently, semipermanently, or retrievable, and it provides a high sampling, real-time measurement with high vertical resolution across the entire length of the fiber itself. In effect, the same “light pipes” that have brought the internet as we know it today have more recently been found to have the amazing sensing capability to make distributed measurements of temperature, strain, and acoustics thanks to its large bandwidth, and data transmission speed. Optical fibers can collect large amounts of data over the length of the fiber with high spatial and temporal resolution while simultaneously transmitting the signal, acting as both the sensing and transmission method.

**Figure 1:**
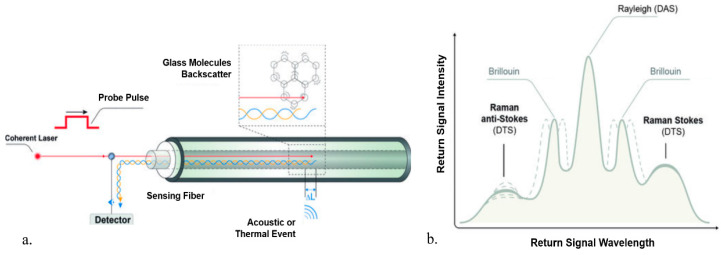
(**a**) Schematic of distributed fiber optic sensing. (**b**) Backscattered signal [6].

A typical set-up for DFOS is shown in Figure 1a [6]. A laser pulse is launched into the optical sensing fiber. As this pulse of light travels down the fiber, interactions with the crystalline structure result in light reflections known as backscatter. A fraction of the scattered light travels back up the optical interrogator, where it is detected and sampled. The backscattered signal consists of the Rayleigh, Brillouin, and Raman bands, as shown in Figure 1b [6]. Typically, Distributed Temperature Sensing (DTS) utilizes the Raman scattering, Distributed Acoustic Sensing (DAS) is measured from Raleigh scattering and Distributed Strain Sensing (DSS) is measured from Brillouin scattering [7].

With the known speed of light in the optical fiber, optical time domain reflectometry (OTDR) principles can be used to determine the point on the fiber from which the light was scattered. This study focuses primarily on the application of Raman-based DTS system in which the temperature is estimated by measuring the relative intensity of the Raman upshifted frequency component (anti-Stokes) and downshifted frequency component (Stokes) [6,7], illustrated in Figure 1b.

Optical fiber sensors can be installed in a wellbore in a permanent, semipermanent, or temporary or retrievable mode [8]. A permanent installation of fiber optics involves cementing the fiber behind the casing. A semipermanent installation can be implemented by clamping the fiber optic line to the tubing, which enables the ability to retrofit existing wells with fiber optic sensor. The temporary or retrievable method is deployed via slick line or coiled tubing [9,10], and is the method used for the CSS test well in this study. There are numerous field examples of successful deployment of distributed fiber optic sensors for injection and production flow profiling, temperature monitoring, seismic measurement, wellbore integrity monitoring, and others [8,9,10,11,12].

### 1.2. Cat Canyon Field Overview

**Figure 2:**
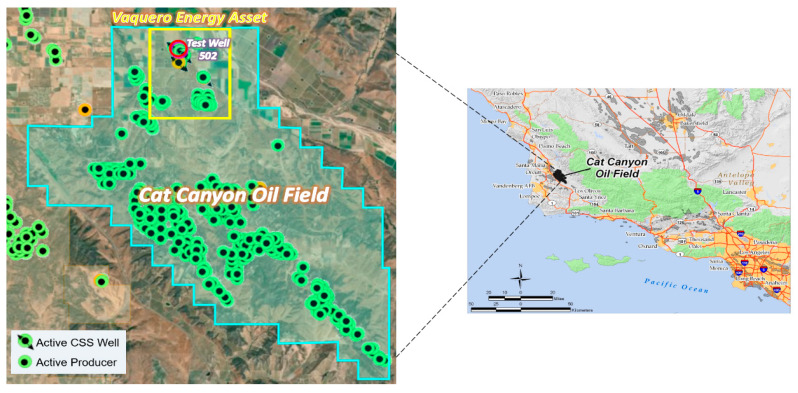
Location of the test well (Ardantz-502) in Vaquero Energy’s asset in the Cat Canyon oil field.

**Figure 3:**
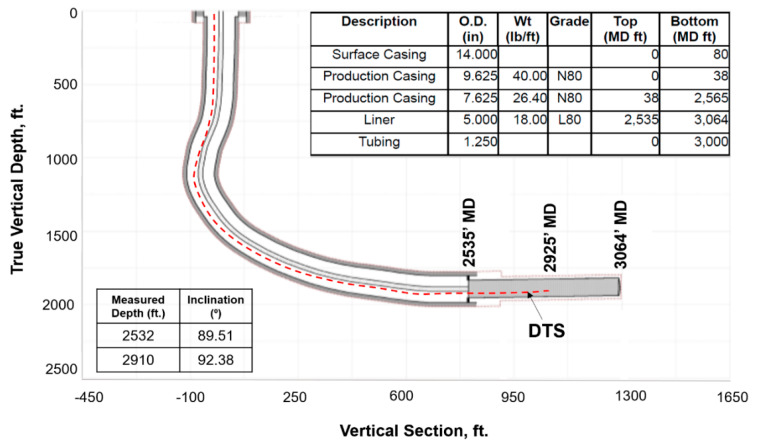
Test well schematic with DTS outside the tubing extending up to 2925 ft.

The data analyzed in this paper was obtained from a CSS operation in the Cat Canyon Oil field located 6 miles east of Santa Maria (California) in the Santa Barbara County. It is a heavy oil field with oil viscosity up to 200,000 cp, at reservoir conditions (105 °F). Vaquero Energy obtained leases in the Cat Canyon field in 2007 and began infill drilling in 2010 to 6 acre spacing to continue the CSS operation. The test well used in this study, Ardantz-502, is located in Vaquero Energy’s asset in Cat Canyon field, as shown in Figure 2. To enable real-time surveillance, DTS was run on a coiled tubing in Ardantz-502, as shown in the well schematic in Figure 3. The horizontal well completion section extends from 2565 ft. to 3064 ft., with a slightly toe-high configuration. The fiber is installed on the exterior of the tubing, but unfortunately only reaching a measured depth of 2925 ft. The well has an open-hole production interval with a premium screen inner liner to help mitigate the sanding issues that gravel packs failed to address in the past.

**Figure 4:**
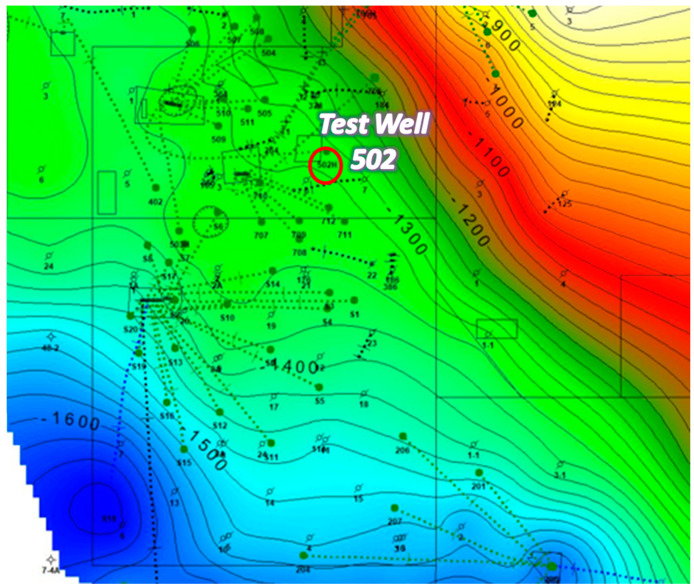
Structural map of the field highlighting the location of the horizontal test well.

**Figure 5:**
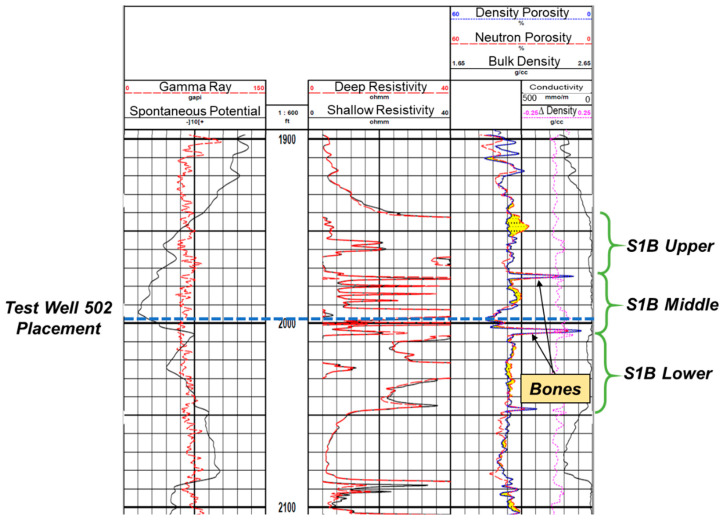
Open hole log from a vertical well adjacent to the test well shows the three lobes in the target sand S1B, separated by calcite hard streaks or bones. Test well 502 is placed at the bottom of the S1B middle lobe.

The location of the test well is highlighted on the structural map of the field shown in Figure 4. The reservoir rock and fluid properties for the target sand, Sisquoc S1B, are summarized in Table 1. The target sand has three distinct lobes, separated by calcite hard streaks or “bones”, as shown in the open hole log in Figure 5 from an adjacent vertical well, Ardantz-712, about 500 ft. from the test well. The bones can be identified from the low neutron porosity and density porosity values in Figure 5.

The test well targets the middle lobe in S1B, and placed at the bottom of the sand interval, as highlighted in Figure 5. The middle lobe appears to be characteristically shaley from the interbedded shale streaks seen in the resistivity logs. There are 42 other CSS wells and a waste water injector in Vaquero Energy’s asset, and the field wide SOR currently averages to about 1. High injection pressures and downhole temperatures (1200 psi and 650 °F) are required to inject the steam. Thus, the main objective of the fiber optic DTS surveillance program was to monitor downhole steam distribution during the CSS operation, to optimize steam usage and production.

## 2. Steam Profiling Using DTS

The primary purpose of flow profiling is to achieve an accurate volumetric allocation of fluid flows between the wellbore and the reservoir. The understanding of downhole steam profile can be critical to verify whether the packers and FCDs are achieving acceptable steam conformance in steam injectors. A thermal model accounts for both geothermal and Joule–Thomson effects as fluid flow is exchanged between the wellbore and the reservoir, as long as there is significant enough thermal contrast between the fluid flows and the surrounding/background environment. Traditional downhole sensing methods are constrained to being implemented during steady-state conditions or single-point dynamic conditions. DTS, on the other hand, provides real-time temperature profiles over the entire length of the well, which does not require sensor or tool movement in the wellbore. This provides a more accurate identification of wellbore temperature and unique insight into temperature transient effects [9]. Measurements from injection, monitoring, and production wells can be combined with surface data, to give a detailed reservoir profile.

DTS has been employed for reservoir monitoring since the 1990s for a variety of applications including SAGD [13,14] water and gas injection [15], horizontal well production [16], oil and gas flow profiling [17], steam breakthrough identification [18], and determination of flow contribution of each zone in a multi-zone reservoir [19]. Some of the common techniques for measuring and interpreting the injection profile with DTS are summarized by [20,21,22,23]. DTS was also used for a steam injection project to identify steam zone development and numerical approach was used to estimate flowrate profile from the temperature profile [24,25]. However, for multiphase flow or a horizontal well, additional data may be required to perform reliable profiling with DTS data. The estimation of fluid injection per zone has traditionally been evaluated with radioactive traces, but DTS is proving to be a suitable candidate as the former technique did not prove to be efficient enough aside from the additional Health, Safety, and Environment (HSE) risks it poses.

In this study, we present a novel technique for estimating steam injection profile by utilizing intelligent optimization techniques to integrate high resolution DTS measurements in numerical simulations. By history matching the temperature profile provided by the DTS measurements during the soak period, a quantitative estimate of the distribution of steam during the injection process can be obtained. Therefore, the automated history matching workflow provided in this paper illustrates how DTS measurements can be utilized for accurate injection profile predictions along the entire injected interval of the CSS well, while honoring the injection, production and temperature history.

## 3. Integration of DTS Data in Reservoir Simulation

Thermal reservoir simulation modeling can be used to estimate the downhole temperature profile by numerically solving the mass balance, energy balance, and dynamic fluid flow equations at each grid block. Numerical results are further conditioned to be consistent with the previous field performance, in a process called history matching. This is a common practice in oil and gas industry in order to have reasonable future predictions. Given that in many real-world scenarios, even water rates for each well are not certain values but only estimated through allocation, the high resolution DTS profile history matching provides a greater degree of certainty that the actual reservoir dynamics are being captured.

The parameters used to create the thermal simulation model in this study are shown in Table 2. CMG^®^’s Flexwell [26] multisegmented wellbore modeling functionality was utilized to enable the calculation of heat and pressure loss along the length of the wellbore. Flexwell models the fluid and heat flow in the wellbore and between the wellbore and the reservoir. Additionally, it can handle multiple tubing strings, cross-flow, phase segregation, FCD’s, and other transient behavior. The wellbore temperature profiles resulting from the Flexwell numerical models are representative of the annulus temperatures in the simulations that were then used to match the DTS field data, as the fiber is in the wellbore annulus (Figure 3).

The fiber optic DTS outputs a high-resolution temperature profile of the well both in space and time with temperature data every 1.43 ft. at 1 min intervals, resulting in a large dataset. Recently, numerical optimization techniques have been increasingly used for assisted (automated) history matching of large data sets [27,28,29].

### 3.1. Intelligent Optimization Algorithm for DTS Integration

**Figure 6:**
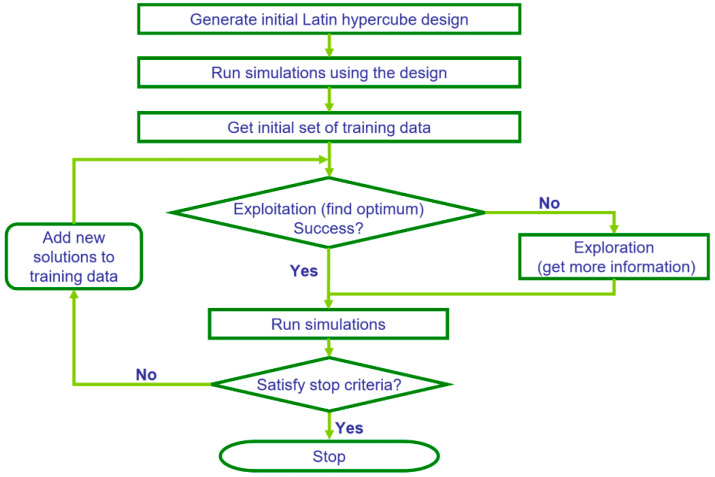
CMG^®^ CMOST Design Exploration Controlled Evolution (DECE) algorithm [30].

In this study, we used CMG CMOST’s Design Exploration Controlled Evolution (DECE), an intelligent optimization algorithm that automatically learns and improves from experience. DECE is a two-stage iterative optimizer, as illustrated in Figure 6 [30]. In the first stage (Design Exploration), the optimizer explores the parameter space and gathers the maximum amount of information about the solution space. In this stage, experimental design techniques are applied to select parameter values and create representative simulation datasets. In the Controlled Evolution stage, statistical analyses are performed for the simulation results obtained in the designed exploration stage. Based on the analyses, the DECE algorithm scrutinizes every candidate value of each parameter to determine if there is a better chance to improve solution quality if certain candidate values are rejected (banned) from being picked again. These rejected candidate values are remembered by the algorithm and they will not be used in the next controlled exploration stage, thus the algorithm continues to learn from its past performance. The input parameters are iterated over the history matching objective functions by continually narrowing their multivariate effects on each other and learning the array of their respective ranges that yield accurate history matched solutions [31]. In other words, the algorithm gets the initial set of training data, finds optimum values that result in matches, adds new solutions to the training data, runs more simulations and, as this process continues, the parameter ranges are shrunk as the algorithm eliminates bad candidate values. The advantages of DECE are that it can handle both continuous and discrete parameters along with hard constraints, and the engine leads to fast and stable convergence without manually having to always change input parameter files.

**Figure 7:**
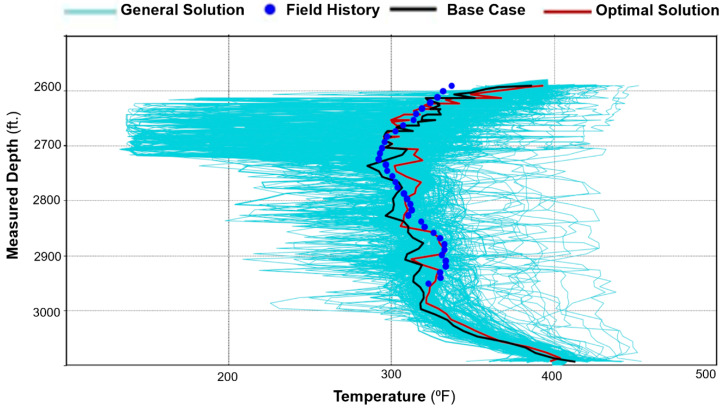
Example illustrating the large number of simulation runs or “experiments” performed and optimized.

The DECE algorithm is ideal for history matching DTS temperature profiles because of its capability to handle the large number of parameters of the collective transmissibility characteristics along the well. As shown in Figure 7, DECE runs thousands of simulations attempting to converge to the objective functions, which in this case is the temperature profile along the well. However, a quality result is only achieved with quality input data. To that end, the workflow presented in this paper provides a framework to provide quality input data for the DECE algorithm by remaining within reasonable ranges for the reservoir and thermal parameters, and maintaining a structured approach in order to capture the transmissibility behavior along the well based on the high resolution temperature data. The next section describes the DECE-assisted history matching workflow to improve the traditional production history matching process by integrating it with the temperature history enabling a more accurate estimation of steam injection profile.

### 3.2. Assisted History Matching Workflow

**Figure 8:**
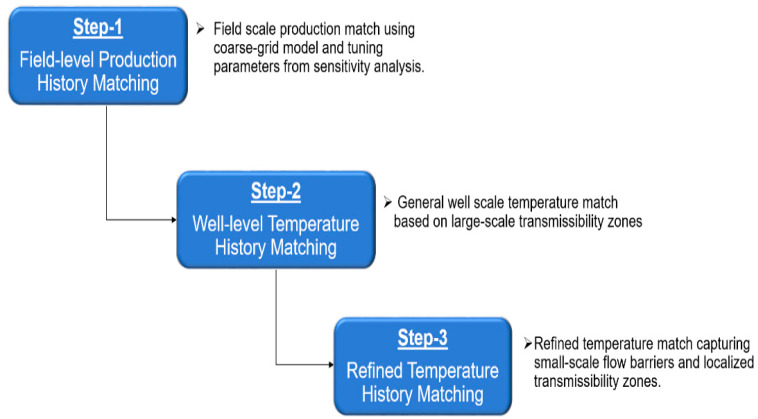
Assisted history matching workflow for integrating production and DTS data for steam profiling.

**Figure 9:**
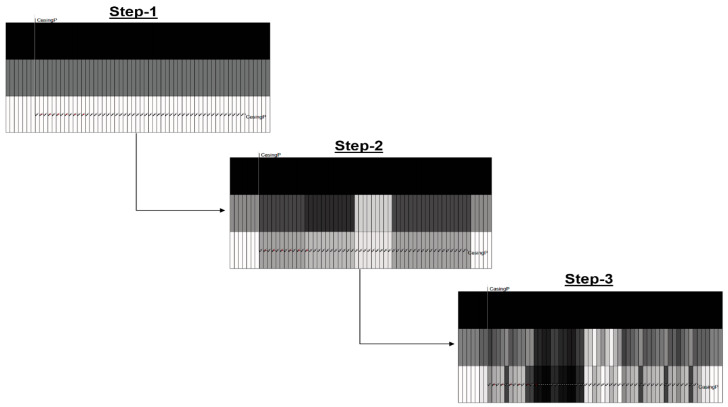
Stepwise grid refinement implemented in each step of the workflow.

A stepwise grid-refinement approach was utilized to optimize computational efficiency and improve overall match quality as illustrated in Figure 8 and Figure 9 and described below.

Step 1: The first step in the workflow consists of a coarse-grid history match of the production history to capture the general reservoir flow dynamics. The tuning parameters in this step are determined from a preliminary sensitivity analysis to evaluate the input variables that have the largest impact on the objective functions and the oil and water rates. The sensitivity analysis enables quality input data for the optimization algorithm to reduce the global error of the desired objective functions based on realistic ranges from the field data. The Response Surface Methodology used in the sensitivity analysis adjusts multiple parameters together and then fits a response surface (polynomial equation) to the results. In effect, the response surface is a proxy for the reservoir simulator that allows fast estimation of the response without having to run thousands of expensive numerical simulations in a CMOST history matching process. Nonlinear parameter relations and cross-terms effect can also be included. Thus, by accounting for both the range of uncertainty provided in the sensitivity analysis combined with specific field knowledge, the DECE algorithm is given suitable input data expedient to convergence while remaining consistent to that particular field. The optimal simulation run that best history matches the production history achieved by the DECE algorithm can then be used as the basis for the general temperature profile history matching in Step 2. Field pressures and gas rates, when available, may also be included for history matching in this step of the workflow.

Step 2: The second step in the workflow includes an additional layer of detail by incorporating the objective function of history matching the temperature profile. This is based on the general knowledge of respective geologic zones along the length of the well in addition to any knowledge of potential flow barriers. Because of the natural geological heterogeneity of real-world formations, there are areas where more injection occurs due to favorable transmissibility characteristics in these zones, and the temperature profile history reflects the injection profile through time. Thus, by dividing the well into large-scale zones as shown in Figure 9, the specific transmissibility characteristics of these zones are incorporated in the DECE algorithm to yield a general temperature profile match while still honoring the production objective functions. In effect, this step of the workflow captures the relative dynamics between the heel, middle, and toe zones using the high-resolution DTS data. This step in the workflow is only executed until a general match of the relative maxima and minima of the field temperature history is achieved before advancing to Step 3, which is based on input parameters that are even more refined in space in order to better history match the temperature profile.

Step 3: The third step in the workflow fully utilizes the DECE algorithm’s ability to handle a large number of parameters by refining the transmissibility zones to even narrower ranges as shown in Figure 9. The number of transmissibility zones depends on the relative amount of temperature change in any particular zone that must be accounted for at a refined scale. The identification of flow barriers is also possible thanks to DECE’s capability to handle discrete parameters enabling the opening and closing of specific perforations within the well to be input parameters. A flow barrier affects the temperature profile much more sharply than simply a reduced transmissibility zone. Therefore, having flow barriers as input parameters for the DECE algorithm enables a more accurate history match since the temperature objective functions are capturing the actual dynamics of flow barrier zones while staying true to the rest of the temperature data along the well.

The completed workflow provides a history match of the production, injection, and temperature profile, with very low global error. Adopting intelligent optimization techniques enables better integration of high resolution DTS data into numerical simulation modeling. Uncertainty of predictions is reduced since more of the actual physics is being captured by history matching the temperature profile along the length of the well. Thus, steam conformance can be more rigorously assessed.

## 4. Results

**Figure 10:**
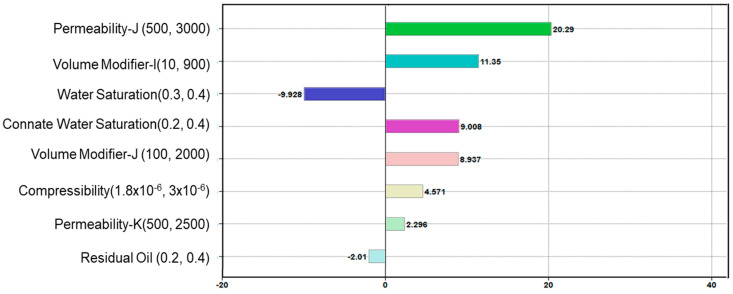
Sensitivity analysis to select tuning parameters with highest impact on production.

Data from two CSS cycles performed on the horizontal test well (Ardantz-502) between January 2018 to March 2019 was analyzed in this study. The DECE-assisted history matching workflow, described in the previous section, was used to match the production (oil and water) and DTS temperature data during those periods to estimate the downhole steam injection profile for both the cycles. Produced gas rates are negligible in this lease, and they are not routinely monitored on a well level. Therefore, gas rates were not included in the analysis. Bottom hole pressure data was also not measured in the field, and likewise not included in the analysis. However, if these data sets are available, it is highly recommended to include them in the history match workflow, as described in Section 3.2. Parameters with high uncertainties and/or variability were included as the history match parameters based on a sensitivity analysis that was conducted to understand their impact on production and temperature as shown in Figure 10. In the simulation model, the I direction is along the wellbore, J is in the lateral direction perpendicular to the wellbore, and K corresponds to the vertical direction. The sensitivity analysis showed that reservoir permeability, water saturation, and volume modifiers (that account for reservoir boundaries) had significant impact on flow rates and temperature. Permeability controls the fluid flow in the reservoir, and consequently has high impact on flow rates of oil, water, gas, and steam, and the corresponding temperature changes. Volume modifiers control the drainage radius and boundary effects that influence the rate of volumetric heating, while water saturation impacts the enthalpy required for heating.

Reservoir heating and fluid flow can be significantly affected by the presence of flow barriers such as shale streaks. The test well (Ardantz-502) is placed at the bottom of the S1B middle lobe, which is ~25 feet thick as shown in Figure 5. The open hole logs from an adjacent well (Ardantz-712) show significant interbedded shale across this zone characterized by the low resistivity values between 1970 to 2000 ft. There are also hard steaks or bones above and below the S1B middle lobe. It is uncertain whether these shale layers and bones will completely restrict flow or act as localized baffles. This is where the DECE algorithm, in the third step of the workflow specifically, was particularly useful by choosing variable transmissibilities in the refined grid model, to account for the localized heterogeneities along the well.

**Figure 11:**
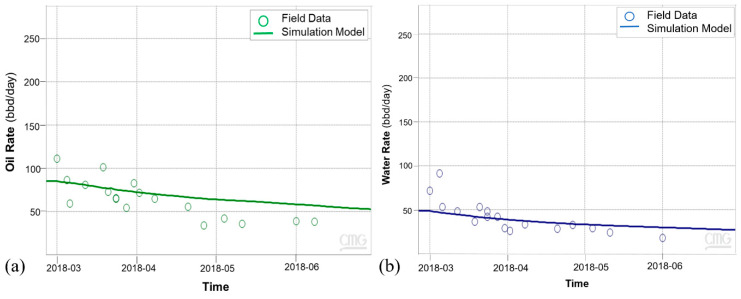
Cycle-1 history match for (**a**) oil rates (**b**) water rates.

**Figure 12:**
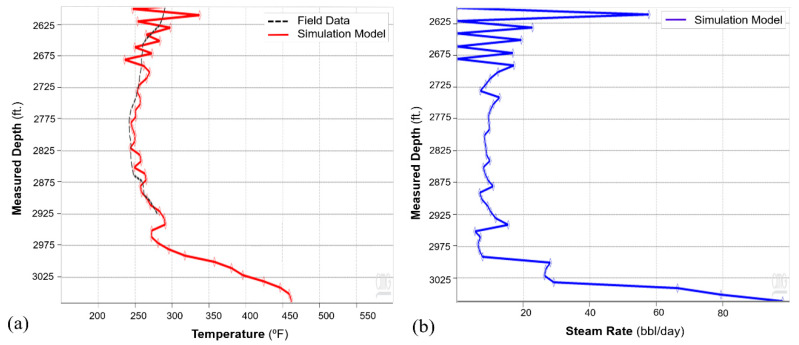
(**a**) Cycle-1 history match of wellbore temperature and field DTS data. (**b**) Steam injection profile for cycle-1.

The history match workflow described in Section 3 was implemented, and Figure 11 shows the resulting oil and water rates for cycle-1, which indicate a reasonable match with the field history. The wellbore temperature and DTS data are compared in Figure 12a. As the fiber only extends up to 2925 ft. (as shown in Figure 3), the DTS data is not available beyond that depth. The history match for the temperature was performed during the soak period. This period was selected because the static temperature data during soak can provide information about steam intake by the different intervals more reliably as compared to temperature data during the injection and production periods, when the temperature is influenced from fluid flow effects. Figure 12a shows a satisfactory match between the wellbore annular temperature from the simulation model and the DTS data. The corresponding steam injection profile is plotted in Figure 12b for the stabilized injection period, which is typically reached after a few days of injection once the transient flow effects ends. The temperature and steam profiles indicate nonuniform reservoir heating with more heat in the heel and the toe sections relative to the rest of the well. As the steam is injected down the tubing, high flow is expected at the heel, which is the first exit point into the reservoir. Further, as the well has a slightly toe-high geometry (Figure 3), high steam flow at the toe is observed due to buoyancy.

**Figure 13:**
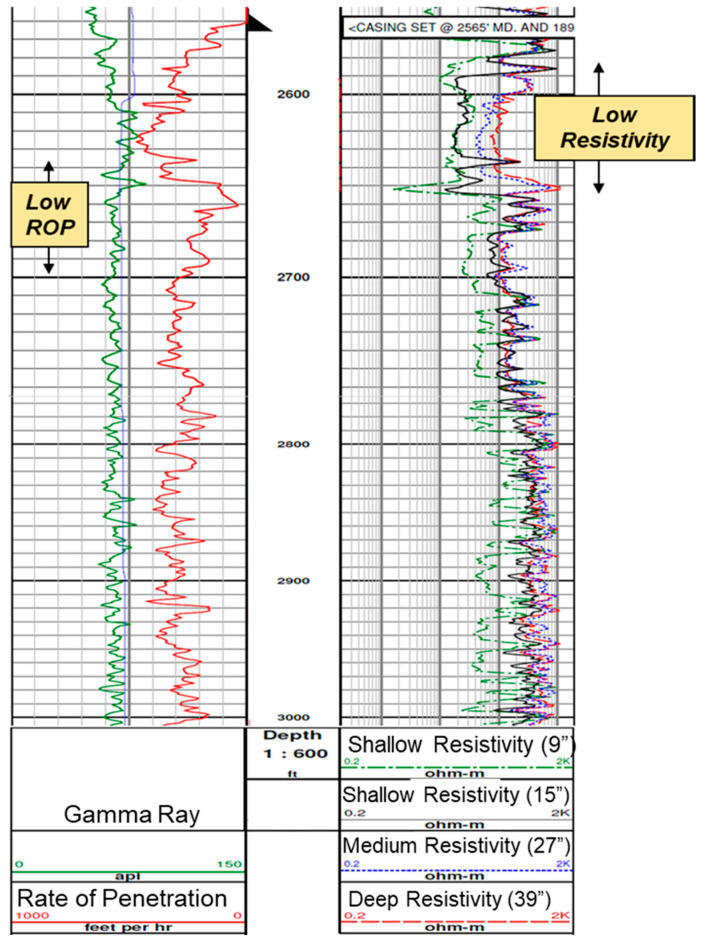
LWD profiles from test well (Ardantz-502) indicate shale baffles above 2650 ft. (characterized by low resistivity) and low ROP observed during drilling likely due to hard streaks above 2700 ft.

The fluctuations in steam and temperature profile above 2700 ft are a result of the geological flow baffles and barriers as indicted by the Logging While Drilling (LWD) logs for the test well as shown in Figure 13. The lower Rate of Penetration (ROP) observed during drilling around the 2650–2700 ft. depth interval also ascertains the presence of hard streaks, which causes variable steam profile in that interval.

**Figure 14:**
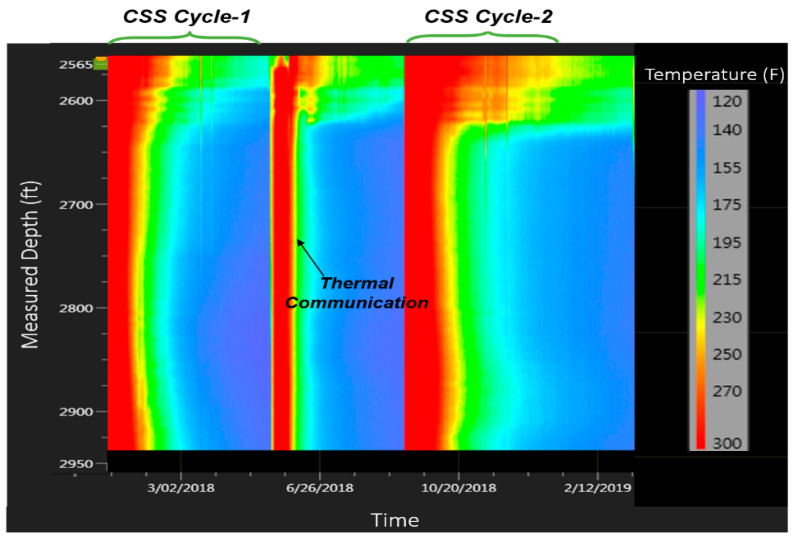
Waterfall chart of DTS field data from test well (Ardantz-502) from January 2018 to March 2019.

**Figure 15:**
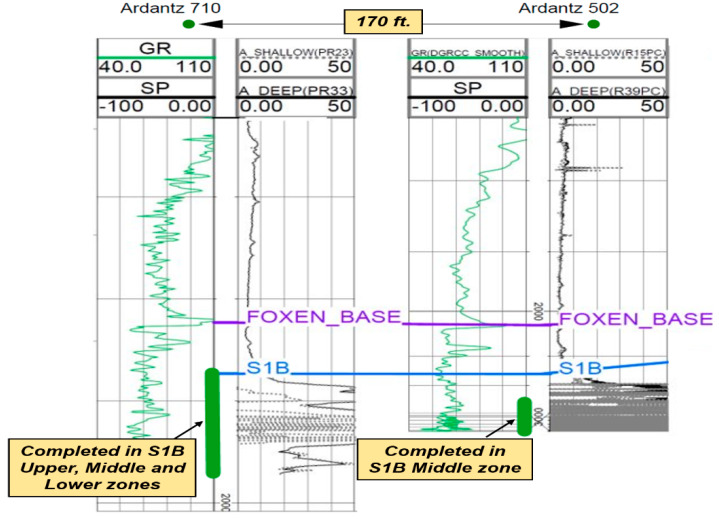
Cross section between Ardantz-502 and Ardantz-710, which are 170 ft. apart (heal to heal distance).

Figure 14 shows a waterfall plot of the entire DTS data during the analysis period from January 2018 to March 2019. The temperature data shows high temperature in May 2018 during which there was no steam injection in the test well. This was an indication of thermal communication with an adjacent well, Ardantz-710, which was being steamed in May 2018. Figure 15 shows the geological cross-section between the two wells, which are only 170 ft. apart (heal to heal distance), with Ardantz-710 slightly downdip of Ardantz-502. Figure 15 shows that both wells target the S1B reservoir; however, Ardantz-710 is completed in S1B upper, middle, and lower zones, whereas the test well (Ardantz-502) is only completed in the S1B-middle zone. Downhole proximity and up-dip structure likely contributed to the steam channeling effect. The thermal communication also potentially contributed to the unfavorable steam profile in the test well during cycle-1, as evident from the high temperatures seen at the toe of Ardantz-502 in Figure 12.

**Figure 16:**
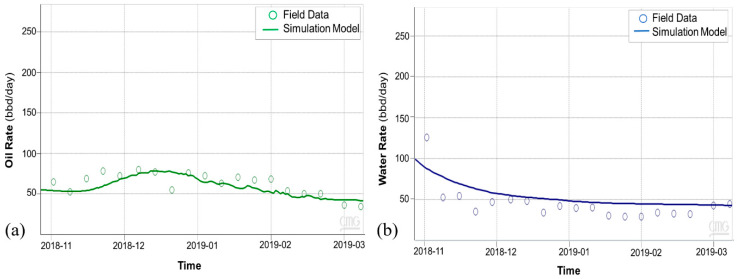
Cycle-2 history match for (**a**) oil rates and (**b**) water rates.

**Figure 17:**
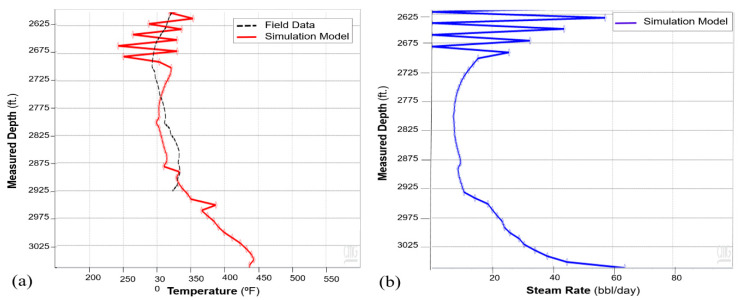
(**a**) Cycle-2 history match of wellbore temperature and field DTS data (**b**) Steam injection profile for cycle-2.

Subsequent to the thermal communication observation, Ardantz-712 was worked over, and the middle zone completion was patched with a cement plug to stop further steam channeling to Ardantz-502, which targets the S1B middle zone. Figure 16 shows the results from the history match of the oil and water rates for CSS cycle-2, performed after the workover of Ardantz-710 indicating decent agreement for oil and water production rates between the simulation and field data. Figure 17a shows satisfactory match between the wellbore temperature from the simulation model and the field DTS data during the soak period of cycle-2. The corresponding steam injection profile, for the stabilized injection period, is shown in Figure 17b. The results indicate less steam going to the toe of the well, as compared to cycle-1 (Figure 12b). This shows that the workover of Ardantz-710 resolved the thermal communication effect to some extent. Similar to cycle-1, fluctuations in steam profile and temperature is seen above 2700 ft as a result of the geological flow baffles and barriers. Overall, cycle-2 shows improvement in steam conformance as compared to cycle-1 with better steam distribution in the middle zone. However, the steam profile remains nonuniform along the well due to the effect of heterogeneity and baffling in the S1B middle lobe apparent from the well logs in Figure 5 and Figure 13. The continuous downhole data from fiber optic surveillance program made it possible to not only detect the thermal communication event in real-time, but also assess the effectiveness of the remedial workover, which could have been missed with conventional logging. The optimization assisted workflow successfully integrates high resolution DTS measurements in numerical simulations, while accounting for reservoir heterogeneity through stepwise grid refinement.

## 5. Conclusions

The novel automated workflow presented in this paper provides a methodology to efficiently integrate high-resolution large DTS datasets in CSS applications, by exploiting intelligent optimization techniques to enable the history matching process in reservoir simulations. The value of including the temperature history, in addition to production history, not only captures the actual reservoir flow dynamics, but also provides insights into the geologic heterogeneity and injection profile variability during a CSS application. Using actual field data from a horizontal well CSS operation in a heavy oil field in California, the value of integrating DTS with production data is illustrated as it allows the injection profile to be accurately estimated along the entire completion interval.

A multisegmented wellbore model was implemented to capture the relative wellbore and reservoir dynamics, which aided in accurately matching the annular temperature provided by the DTS. The key to expediently utilizing the workflow provided is carrying out a stepwise grid-refinement approach, which optimizes computational efficiency and improves predictive accuracy. The proxy models created from evolutionary optimization techniques streamlined the workflow and also helped quantify the uncertainty in the predictive models due to the reservoir heterogeneity.

The history matched results for the two respective cycles in this study resulted in unique injection profiles that can be differentiated due to thermal communication being present in one cycle. The distinction between the relative dynamics at the heel, middle, and toe for the respective cycles with and without thermal communication is clear and can be noted in definite signatures. The effectiveness of remedial workover of the communicating well can also be assessed from the subsequent DTS profile. Thus, the downhole thermal communication was both qualitatively and quantitatively captured by implementing the workflow presented here demonstrating the benefit of real-time continuous data from fiber optic surveillance as compared to conventional monitoring.

## Figures and Tables

**Table 1:** Sisquoq S1B reservoir rock and fluid properties.

Parameters	Value
Porosity [%]	33
Oil Saturation [%]	50–65
Permeability [mD]	1000–2000
API Gravity	5.5–10.1
GOR [scf/bbl]	470
Reservoir Temperature [°F]	105
Sour Gas Concentration [ppm]	1100
Oil Formation Volume Factor	1.03
Oil Viscosity @ 100 °F [cp]	200,000

**Table 2:** Simulation model and Flexwell input parameters.

**Parameters**	
Number of Grid Blocks I, J, K	630, 230, 30
Grid Block Dimensions: dx, dy, dz [ft]	10, 10, 10
Max Production Constraint [bbd]	2000
Steam Injection Rate [bbl/d]	1100
CSS Cycle Injection Duration [days]	9
CSS Cycle Soak Duration [days]	3
**Flexwell Parameters**	
Wall Heat Capacity [BTU/(ft3 × F)]	487.5
Wall Heat Conductivity [BTU/(ft × day × F)]	350
Cement Heat Capacity [BTU/(ft3 × F)]	27.56
Cement Heat Conductivity [BTU/(ft × day × F)]	18.96
Relative Roughness	0.0001
Maximum Nusselt Number	10,000
Wall ID [ft]	0.581
Wall OD [ft]	0.635
